# Hypersexuality and Emotional Congruence with Children in Individuals with Pedophilia and Hebephilia: A Comparison of Implicit and Explicit Assessment Methods

**DOI:** 10.1192/j.eurpsy.2026.12114

**Published:** 2026-07-31

**Authors:** P. Heindl, M. Zwißler, T. Donhauser, S. Schobel, K. Fischer, L. Löhrs, J. Moussiopoulou, M. Wertz, K. Schiltz

**Affiliations:** Department of Forensic Psychiatry, University Hospital, Ludwig Maximilians University Munich, Munich, Germany

## Abstract

**Introduction:**

Implicit and explicit measurement methods play a central role in assessing atypical sexual interests, including pedo-hebephilia. Among implicit approaches, viewing time (VT) paradigms are particularly prevalent, as response latencies to sexual stimuli serve as indirect indicators of sexual interest. Nevertheless, it remains unclear to what extent additional factors related to sexual responsivity and emotional processing—such as hypersexuality or emotional congruence with children (ECC)—may affect the results obtained from both implicit and explicit assessment methods.

**Objectives:**

The aim of this study was to examine the influence of hypersexuality and ECC on implicit (VT) and explicit measures (attractiveness ratings) methods among pedophilic, hebephilic and teleiophilic men.

**Methods:**

Attractiveness ratings and VT were assessed on a computer. To record VT, eight images for each sex across all five Tanner stages were presented, resulting in a total of 80 trials. Participants rated the attractiveness of the images on a 5-point Likert scale (1 = “not sexually attractive,” 5 = “very sexually attractive”) by pressing a key while their response latencies (VT) were simultaneously recorded without their awareness. For data analysis, pictures corresponding to Tanner stages 1–3 were classified as child stimuli, whereas Tanner stages 4–5 were classified as adult stimuli. Sexual preference was determined through a clinical interview, and hypersexuality and ECC were collected using standardized questionnaires (HBI-19, EKK-R).

**Results:**

The total sample consisted of men with pedophilic (n = 34) and hebephilic interests (n = 25) as well as a teleiophilic control group (n = 53). Results showed significantly longer VT and higher attractiveness ratings for child stimuli in pedophilic and hebephilic participants. In the total sample, but not within individual groups, hypersexuality was associated with longer VT and higher attractiveness ratings for child stimuli. Within the clinical groups, hypersexuality was a significant predictor of attractiveness ratings for child stimuli. ECC was associated with VT only at the total sample level. No interaction effects between ECC, hypersexuality, and sexual preference were found.

**Conclusions:**

The findings indicate that potentially confounding variables such as hypersexuality and ECC might systematically affect the diagnostic validity of both implicit and explicit preference measures.

**Disclosure of Interest:**

None Declared

